# Binomial difference sequence spaces of fractional order

**DOI:** 10.1186/s13660-018-1873-x

**Published:** 2018-10-10

**Authors:** Jian Meng, Liquan Mei

**Affiliations:** School of Mathematics and Statistics, Xi’an Jiaotong University, Xi’an, P.R. China

**Keywords:** 46A45, 46B45, 46B50, Sequence space, Matrix transformation, Fractional difference operator, *α*-, *β*-, and *γ*-duals

## Abstract

In this paper, we introduce the sequence spaces $b^{r,s}_{0}( \nabla^{(\alpha)})$, $b^{r,s}_{c}(\nabla^{(\alpha)})$, and $b^{r,s}_{\infty }(\nabla^{(\alpha)})$. We investigate some functional properties, inclusion relations, and the *α*-, *β*-, *γ*-, and continuous duals of these sets.

## Introduction

Let *w*, $\ell_{p}$, $\ell_{\infty }$, *c*, and $c_{0}$ denote the spaces of all, *p*-absolutely summable, bounded, convergent, and null sequences $x=(x_{k})$ with complex terms $x_{k}$, respectively, where $1\leq p<\infty $ and $k\in \mathbb{N}=\{0,1,2,\ldots\}$. A sequence space *X* is called a *BK*-space if it is a Banach space with continuous coordinates $p_{k}:X\rightarrow \mathbb{C}$ defined by $p_{k}(x)=x _{k}$ for $x=(x_{k})\in X$ and $k\in \mathbb{N}$. The most important result of the theory of *BK*-spaces is that matrix mappings between *BK*-spaces are continuous [13]. The sequence spaces $\ell_{\infty }$, *c*, and $c_{0}$ with their sup-norm are *BK*-spaces.

The concept of a difference sequence space was firstly introduced by Kizmaz [22] by defining the set $Z(\Delta)=\{x=(x_{k}):( \Delta x_{k})\in Z\}$ for $Z\in \{\ell_{\infty },c,c_{0}\}$, where $\Delta x_{k}=x_{k}-x_{k+1}$ for $k\in \mathbb{N}$. The idea of a difference sequence was generalized by Et and Çolak [14–16] by defining the spaces $Z(\Delta^{m})=\{x=(x_{k}):(\Delta^{m} x_{k}) \in Z\}$ for $Z\in \{\ell_{\infty },c,c_{0}\}$, where $m\in \mathbb{N}$, $\Delta^{m} x_{k}=\Delta^{m-1}x_{k}-\Delta^{m-1}x_{k+1}$ for $k\in \mathbb{N}$. For a positive proper fraction *α*, Baliarsingh and Dutta [4, 5] defined the fractional difference operator $\Delta^{\alpha }$ by

$$\begin{aligned} \Delta^{\alpha }x_{k}=\sum_{i=0}^{\infty }(-1)^{i} \frac{\Gamma ( \alpha +1)}{i!\Gamma (\alpha -i+1)}x_{k+i} \end{aligned}$$

for $k\in \mathbb{N}$, where the Euler gamma function $\Gamma (p)$ of a real number *p* with $p\notin \{0,-1,-2, -3,\ldots\}$ can be expressed as an improper integral $\Gamma (p)=\int_{0}^{\infty }e^{-t}t^{p-1}\,dt$. It is observed that

(i) $\Gamma (p+1)=p!$ for $p\in \mathbb{N}$,
(ii) $\Gamma (p+1)=p\Gamma (p)$ for $p\in \mathbb{R}\setminus \{0,-1,-2,-3,\ldots\}$.

Some definitions of fractional derivatives have been generalized by using a set of new difference sequence spaces of fractional order [3]. Application of fractional derivatives becomes more apparent in diffusion processes, modeling mechanical systems, and many other fields.

Let *X*, *Y* be two sequence spaces, and let $A=(a_{n,k})$ be an infinite matrix with complex numbers $a_{n,k}$, $n,k\in \mathbb{N}$. Let the sequence space $X_{A}$ defined by $X_{A}=\{x=(x_{k}):Ax \in X\}$ denote the domain of matrix *A* in the space *X*, where $Ax=\{(Ax)_{n} \}$, the *A*-transform of *x*, is defined by $(Ax)_{n}=\sum_{k=0}^{ \infty }a_{n,k}x_{k}$, $n\in \mathbb{N}$. Let $(X : Y)$ denote the class of all matrices such that $X \subseteq Y_{A}$. The matrix domain approach has been employed by Başarir and Kara [6–10], Kara and İlkhan [19–21], Polat and Başar [26], Song and Meng [23–25, 27], and many others to introduce new sequence spaces.

The Euler sequence spaces $e^{r}_{0}$, $e^{r}_{c}$, and $e^{r}_{ \infty }$ were defined by Altay and Başar [1] and Altay, Başar, and Mursaleen [2]. Moreover, Kadak and Baliarsingh [18] introduced a generalized Euler mean difference operator $E^{r}(\nabla^{(\alpha)})$ of fractional order, where the matrix $\nabla^{(\alpha)}=(\nabla^{(\alpha)}_{n,k})$ is defined by

$$ \nabla^{(\alpha)}_{n,k}= \textstyle\begin{cases} (-1)^{n-k}\frac{\Gamma (\alpha +1)}{(n-k)!\Gamma (\alpha -n+k+1)} & \text{if } 0\leq k\leq n, \\ 0 & \text{if }k>n. \end{cases} $$

Let $r,s\in \mathbb{R}$ and $r+s\neq 0$. Then the binomial matrix $B^{r,s}=(b_{n,k}^{r,s})$ is defined by

$$b_{n,k}^{r,s}=\left\{ \begin{array}{cc} \frac{1}{{\left(s+r\right)}^{n}}\left(\begin{array}{c} n\\ k \end{array}\right)s^{n-k}r^{k} & \text{if }0\leq k\leq n,\\ 0 & \text{if }k>n, \end{array}\right.$$

for all $k,n\in \mathbb{N}$. If $s+r =1$, then we obtain the Euler matrix $E^{r}$. Bişgin [11, 12] defined the binomial sequence spaces

$$\begin{array}{c} b_{0}^{r,s}=\left\{x=(x_{k}):\lim_{n\to \infty }\frac{1}{{\left(s+r\right)}^{n}}\sum_{k=0}^{n}\left(\begin{array}{c} n\\ k \end{array}\right)s^{n-k}r^{k}x_{k}=0\right\},\\ b_{c}^{r,s}=\left\{x=(x_{k}):\lim_{n\to \infty }\frac{1}{{\left(s+r\right)}^{n}}\sum_{k=0}^{n}\left(\begin{array}{c} n\\ k \end{array}\right)s^{n-k}r^{k}x_{k}\text{ exists}\right\}, \end{array}$$

and

$$b_{\infty }^{r,s}=\left\{x=(x_{k}):\underset{n\in N}{\sup}\left|\frac{1}{{\left(s+r\right)}^{n}}\sum_{k=0}^{n}\left(\begin{array}{c} n\\ k \end{array}\right)s^{n-k}r^{k}x_{k}\right|<\infty \right\}.$$

The purpose of this paper is to generalize the sequence spaces $e^{r}_{0}(\nabla^{(\alpha)})$, $e^{r}_{c}(\nabla^{(\alpha)})$, and $e^{r}_{\infty }(\nabla^{(\alpha)})$ and introduce the binomial difference sequence spaces $b^{r,s}_{0}(\nabla^{(\alpha)})$, $b^{r,s}_{c}(\nabla^{(\alpha)})$, and $b^{r,s}_{\infty }( \nabla^{(\alpha)})$ of fractional order whose $\nabla^{(\alpha)}$-transforms are in the spaces $b^{r,s}_{0}$, $b^{r,s}_{c}$, and $b^{r,s}_{\infty }$. These new sequence spaces are generalizations of the spaces defined in [11, 12, 18, 23, 25, 26].

## Difference sequence spaces of fractional order

In this chapter, we introduce the binomial difference sequence spaces $b^{r,s}_{0}(\nabla^{(\alpha)})$, $b^{r,s}_{c}(\nabla^{(\alpha)})$, and $b^{r,s}_{\infty }(\nabla^{(\alpha)})$ of fractional order and investigate some functional properties and inclusion relations.

Define the spaces $b^{r,s}_{0}(\nabla^{(\alpha)})$, $b^{r,s}_{c}( \nabla^{(\alpha)})$, and $b^{r,s}_{\infty }(\nabla^{(\alpha)})$ by

$$Z\bigl(\nabla^{(\alpha)}\bigr)=\bigl\{ x=(x_{k}): \nabla^{(\alpha)} (x_{k})\in Z\bigr\} $$

for $Z\in \{b^{r,s}_{0}, b^{r,s}_{c}, b^{r,s}_{\infty }\}$. By taking the $\nabla^{(\alpha)}$-transform of *x* in the spaces $b^{r,s}_{0}$, $b^{r,s}_{c}$, and $b^{r,s}_{\infty }$ the spaces $b^{r,s}_{0}( \nabla^{(\alpha)})$, $b^{r,s}_{c}(\nabla^{(\alpha)})$, and $b^{r,s}_{\infty }(\nabla^{(\alpha)})$ can be redefined by

2.1 $$\begin{aligned} b^{r,s}_{0}\bigl(\nabla^{(\alpha)}\bigr)= \bigl(b^{r,s}_{0}\bigr)_{\nabla^{(\alpha)}},\qquad b ^{r,s}_{c}\bigl(\nabla^{(\alpha)}\bigr)= \bigl(b^{r,s}_{c}\bigr)_{\nabla^{(\alpha)}},\qquad b ^{r,s}_{\infty }\bigl(\nabla^{(\alpha)}\bigr)= \bigl(b^{r,s}_{\infty }\bigr)_{ \nabla^{(\alpha)}}. \end{aligned}$$

The sequence spaces $b^{r,s}_{0}(\nabla^{(\alpha)})$, $b^{r,s}_{c}( \nabla^{(\alpha)})$, and $b^{r,s}_{\infty }(\nabla^{(\alpha)})$ include some particular cases in certain cases of *s*, *r*, and *α*.

(i) For $\alpha =0$, these sequence spaces generalize the spaces $b^{r,s}_{0} $, $b^{r,s}_{c} $, and $b^{r,s}_{\infty }$ defined by Bişgin [11, 12].
(ii) For $s+r=1$, these sequence spaces generalize the spaces $e^{r}_{0}(\nabla^{(\alpha)})$, $e^{r}_{c}(\nabla^{(\alpha)})$, and $e^{r}_{\infty }(\nabla^{(\alpha)})$ defined by Kadak and Baliarsingh [18].
(iii) For $\alpha =m\in \mathbb{N}$, these sequence spaces generalize the spaces $b^{r,s}_{0}(\nabla^{(m)})$, $b^{r,s}_{c}( \nabla^{(m)})$, and $b^{r,s}_{\infty }(\nabla^{(m)})$ defined by Meng and Song [23].
(iv) For $s+r=1$ and $\alpha =m\in \mathbb{N}$, these sequence spaces generalize the spaces $e^{r}_{0}(\nabla^{(m)}) $, $e^{r}_{c}( \nabla^{(m)})$, and $e^{r}_{\infty }(\nabla^{(m)}) $ defined by Polat and Başar [26].

Define the sequence $y=(y_{n})$ by the $B^{r,s}(\nabla^{(\alpha)})$-transform of a sequence $x=(x_{k})$, that is,

2.2 $$\begin{array}{rcl} y_{n} & = & {\left[B^{r,s}\left(\nabla^{\left(\alpha \right)}\right)(x_{k})\right]}_{n}\\ & = & \frac{1}{{\left(s+r\right)}^{n}}\sum_{k=0}^{n}\sum_{i=k}^{n}{\left(-1\right)}^{i-k}\left(\begin{array}{c} n\\ i \end{array}\right)\frac{\Gamma (\alpha +1)}{\left(i-k)!\Gamma (\alpha -i+k+1\right)}s^{n-i}r^{i}x_{k} \end{array}$$

for each $n\in \mathbb{N}$.

### Theorem 2.1

*Let* $Z\in \{b^{r,s}_{0}, b^{r,s}_{c}, b^{r,s}_{\infty }\}$. *Then* $Z(\nabla^{(\alpha)})$ *are* *BK*-*spaces with the norm* $\| x \|_{Z(\nabla^{(\alpha)})}=\| \nabla^{(\alpha)} (x_{k}) \|_{Z}$.

### Proof

Theorem 2.1 of Bişgin [11, 12] and Theorem 4.3.12 of Wilansky [29] imply that the spaces $Z(\nabla^{(\alpha)})$ are *BK*-spaces. □

### Theorem 2.2

*The inclusion* $b^{r,s}_{0}(\nabla^{(\alpha)})\subseteq b^{r,s}_{c}( \nabla^{(\alpha)})\subseteq b^{r,s}_{\infty }(\nabla^{(\alpha)})$ *is strict*.

### Proof

Proof follows from Lemma 2.3 of Et and Nuray [17]. □

### Theorem 2.3

*The inclusions* $e_{0}^{r}(\nabla^{(\alpha)})\subseteq b^{r,s}_{0}( \nabla^{(\alpha)})$, $e_{c}^{r}(\nabla^{(\alpha)})\subseteq b^{r,s} _{c}(\nabla^{(\alpha)})$, *and* $e_{\infty }^{r}(\nabla^{(\alpha)}) \subseteq b^{r,s}_{\infty }(\nabla^{(\alpha)})$ *are strict*.

### Proof

We only give the proof of the inclusion $e_{0}^{r}(\nabla^{(\alpha)}) \subseteq b^{r,s}_{0}(\nabla^{(\alpha)})$. The others can be proved similarly.

It is clear that $e_{0}^{r}(\nabla^{(\alpha)})\subseteq b^{r,s}_{0}( \nabla^{(\alpha)})$. Further, to show that this inclusion is strict, let $0< r<1$ and $s=4$ and define the sequence $x=(x_{k})$ by

$$\begin{aligned} x_{k}=\sum_{j=0}^{k}(-1)^{k-j} \frac{\Gamma (-\alpha +1)}{(k-j)!\Gamma (-\alpha -k+j+1)}\biggl(-\frac{3}{r}\biggr)^{j} \end{aligned}$$

for $k\in \mathbb{N}$. We have $[E^{r}(\nabla^{(\alpha)})( x_{k})]_{n}=((-2-r)^{n}) \notin c_{0}$ and $[B^{r,s}(\nabla^{(\alpha)})( x_{k})]_{n}=(( \frac{1}{4+r})^{n})\in c_{0}$. Therefore, the inclusion $e_{0}^{r}( \nabla^{(\alpha)})\subseteq b^{r,s}_{0}(\nabla^{(\alpha)})$ is strict. □

### Theorem 2.4

*The spaces* $b^{r,s}_{0}(\nabla^{(\alpha)})$, $b^{r,s}_{c}( \nabla^{(\alpha)})$, *and* $b^{r,s}_{\infty }(\nabla^{(\alpha)})$ *are linearly isomorphic to the spaces* $c_{0}$, *c*, *and* $\ell_{\infty }$, *respectively*.

### Proof

We prove the theorem only for the space $b^{r,s}_{0}(\nabla^{(\alpha)})$. To prove $b^{r,s}_{0}(\nabla^{(\alpha)})\cong c_{0}$, we will show the existence of a linear bijection between the spaces $b^{r,s}_{0}(\nabla^{(\alpha)})$ and $c_{0}$.

Let us denote the transformation $T:b^{r,s}_{0}(\nabla^{(\alpha)}) \rightarrow c_{0}$ by $T(x)=B^{r,s}(\nabla^{(\alpha)})( x_{k})$. The linearity of *T* is clear, and $x=0$ whenever $T(x)=0$. Hence *T* is injective.

Let $y=(y_{n})\in c_{0} $ and define the sequence $x=(x_{k})$ by

2.3 $$x_{k}=\sum_{i=0}^{k}{\left(s+r\right)}^{i}\sum_{j=i}^{k}{\left(-1\right)}^{k-j}\left(\begin{array}{c} j\\ i \end{array}\right)\frac{\Gamma (-\alpha +1)}{\left(k-j)!\Gamma (-\alpha -k+j+1\right)}r^{-j}{\left(-s\right)}^{j-i}y_{i}$$

for $k\in \mathbb{N}$. Then we have

$$\lim_{n\to \infty }{\left[B^{r,s}\left(\nabla^{\left(\alpha \right)}\right)(x_{k})\right]}_{n}=\lim_{n\to \infty }\frac{1}{{\left(s+r\right)}^{n}}\sum_{k=0}^{n}\left(\begin{array}{c} n\\ k \end{array}\right)s^{n-k}r^{k}\left(\nabla^{\left(\alpha \right)}\right)(x_{k})=\lim_{n\to \infty }y_{n}=0,$$

which implies that $x\in b^{r,s}_{0}(\nabla^{(\alpha)} )$. Therefore, we obtain that *T* is surjective and norm preserving. This completes the proof. □

We shall construct the Schauder bases for the sequence spaces $b^{r,s}_{0}(\nabla^{(\alpha)})$ and $b^{r,s}_{c}(\nabla^{(\alpha)})$. Because the isomorphism *T* between $b^{r,s}_{0}(\nabla^{(\alpha)})$ and $c_{0}$ (or between $b^{r,s}_{c}(\nabla^{(\alpha)})$ and *c*) is onto, the inverse image of the basis of the space $c_{0}$ (or *c*) is the basis of the space $b^{r,s}_{0}(\nabla^{(\alpha)})$ (or $b^{r,s}_{c}(\nabla^{(\alpha)})$). For $k\in \mathbb{N}$, define the sequence $g^{(k)}(r,s)=\{g^{(k)}_{i}(r,s)\}_{i \in \mathbb{N}}$ by

$$g_{i}^{\left(k\right)}(r,s)=\left\{ \begin{array}{cc} 0 & \text{if }0\leq i<k,\\ {\left(s+r\right)}^{k}\sum_{j=k}^{i}{\left(-1\right)}^{i-j}\left(\begin{array}{c} j\\ k \end{array}\right)\frac{\Gamma (-\alpha +1)}{\left(i-j)!\Gamma (-\alpha -i+j+1\right)}r^{-j}{\left(-s\right)}^{j-k} & \text{if }i\geq k. \end{array}\right.$$

### Theorem 2.5

*The sequence* $(g^{(k)}(r,s))_{k\in \mathbb{N}}$ *is the Schauder basis for the space* $b_{0}^{r,s}(\nabla^{(\alpha)})$, *and every* *x* *in* $b_{0}^{r,s}(\nabla^{(\alpha)})$ *has a unique representation by*

2.4 $$\begin{aligned} x=\sum_{k} \lambda_{k}(r,s) g^{(k)}(r,s), \end{aligned}$$

*where* $\lambda_{k}(r,s)= [B^{r,s}(\nabla^{(\alpha)})( x_{i})]_{k}$ *for* $k\in \mathbb{N}$.

### Theorem 2.6

*Define the sequence* $g=(g_{n})$ *by*

$$g_{n}=\sum_{k=0}^{n}{\left(s+r\right)}^{k}\sum_{j=k}^{n}{\left(-1\right)}^{n-j}\left(\begin{array}{c} j\\ k \end{array}\right)\frac{\Gamma (-\alpha +1)}{\left(n-j)!\Gamma (-\alpha -n+j+1\right)}r^{-j}{\left(-s\right)}^{j-k}$$

*for* $n\in \mathbb{N}$ *and* $\lim_{k\rightarrow \infty }\lambda_{k}(r,s)=l$. *The set* $\{g, g^{(0)}(r,s), g^{(1)}(r,s),g^{(2)}(r,s),\ldots\}$ *is the Schauder basis for the space* $b_{c}^{r,s}(\nabla^{(\alpha)})$, *and every* *x* *in* $b_{c}^{r,s}(\nabla^{(\alpha)})$ *has a unique representation by*

2.5 $$\begin{aligned} x=lg+\sum_{k} \bigl[\lambda_{k}(r,s)-l \bigr] g^{(k)}(r,s). \end{aligned}$$

## The *α*-, *β*-, *γ*-, and continuous duals

In this section, we determine the *α*-, *β*-, *γ*-, and continuous duals of the spaces $b_{0}^{r,s}(\nabla^{(\alpha)})$, $b_{c}^{r,s}(\nabla^{(\alpha)})$, and $b_{\infty }^{r,s}( \nabla^{(\alpha)})$.

For two sequence spaces *X* and *Y*, the set $M(X,Y)$ is defined by

$$\begin{aligned} M(X,Y)=\bigl\{ u=(u_{k}):ux=(u_{k}x_{k})\in Y \text{ for all } x=(x_{k}) \in X\bigr\} . \end{aligned}$$

Let *bs* and *cs* denote the sequence spaces of all bounded and convergent series, respectively. In particular,

$$\begin{aligned} X^{\alpha }=M(X,\ell_{1}),\qquad X^{\beta }=M(X,cs), \quad \text{and}\quad X^{ \gamma }=M(X, bs) \end{aligned}$$

are called the *α*-, *β*-, and *γ*-duals of the sequence space *X*, respectively. The space of all bounded linear functionals on *X* denoted by $X^{*}$ is called the continuous dual of the space *X*.

Let us give the following properties needed in Lemma 3.1:

3.1 $$\begin{aligned}& \sup_{K\in \Gamma } \sum_{n} \biggl\vert \sum_{k\in K} a_{n,k} \biggr\vert < \infty, \end{aligned}$$

3.2 $$\begin{aligned}& \sup_{n\in \mathbb{N}} \sum_{k} \vert a_{n,k} \vert < \infty, \end{aligned}$$

3.3 $$\begin{aligned}& \lim_{n\rightarrow \infty }a_{n,k}=a_{k}\quad \text{for } k \in \mathbb{N}, \end{aligned}$$

3.4 $$\begin{aligned}& \lim_{n\rightarrow \infty }\sum_{k}a_{n,k}=a, \end{aligned}$$

3.5 $$\begin{aligned}& \lim_{n\rightarrow \infty }\sum_{k} \vert a_{n,k} \vert =\sum_{k} \Bigl\vert \lim_{n\rightarrow \infty }a_{n,k} \Bigr\vert , \end{aligned}$$

where Γ is the collection of all finite subsets of $\mathbb{N}$.

### Lemma 3.1

([28])

*Let* $A=(a_{n,k})$ *be an infinite matrix*. *Then*

(i) $A\in (c_{0}:\ell_{1})=(c:\ell_{1})=(\ell_{\infty }:\ell _{1})$ *if and only if* (3.1) *holds*.
(ii) $A\in (c_{0}:c)$ *if and only if* (3.2) *and* (3.3) *hold*.
(iii) $A\in (c:c)$ *if and only if* (3.2), (3.3), *and* (3.4) *hold*.
(iv) $A\in (\ell_{\infty }:c)$ *if and only if* (3.3) *and* (3.5) *hold*.
(v) $A\in (c_{0}:\ell_{\infty })=(c:\ell_{\infty })=(\ell_{ \infty }:\ell_{\infty })$ *if and only if* (3.2) *holds*.

### Theorem 3.2

*We have* $[b_{0}^{r,s}(\nabla^{(\alpha)})]^{\alpha }=[b_{c}^{r,s}( \nabla^{(\alpha)})]^{\alpha }=[b_{\infty }^{r,s}(\nabla^{(\alpha)})]^{ \alpha }=U^{r,s}_{1}$, *where*

$$\begin{array}{rcl} U_{1}^{r,s} & = & \{u=(u_{k}):\underset{K\in \Gamma }{\sup}\sum_{k}|\sum_{i\in K}{\left(s+r\right)}^{i}\sum_{j=i}^{k}{\left(-1\right)}^{k-j}\left(\begin{array}{c} j\\ i \end{array}\right)\\ & & \times \frac{\Gamma (-\alpha +1)}{\left(k-j)!\Gamma (-\alpha -k+j+1\right)}r^{-j}{\left(-s\right)}^{j-i}u_{k}|<\infty \}. \end{array}$$

### Proof

We immediately derive by (2.3) that

$$u_{k}x_{k}=\sum_{i=0}^{k}{\left(s+r\right)}^{i}\sum_{j=i}^{k}{\left(-1\right)}^{k-j}\left(\begin{array}{c} j\\ i \end{array}\right)\frac{\Gamma (-\alpha +1)}{\left(k-j)!\Gamma (-\alpha -k+j+1\right)}r^{-j}{\left(-s\right)}^{j-i}u_{k}y_{i}={\left(G^{r,s}y\right)}_{k}$$

for $k\in \mathbb{N}$, where $G^{r,s}=(g^{r,s}_{k,i})$ is defined by

$$g_{k,i}^{r,s}=\left\{ \begin{array}{cc} {\left(s+r\right)}^{i}\sum_{j=i}^{k}{\left(-1\right)}^{k-j}\left(\begin{array}{c} j\\ i \end{array}\right)\frac{\Gamma (-\alpha +1)}{\left(k-j)!\Gamma (-\alpha -k+j+1\right)}r^{-j}{\left(-s\right)}^{j-i}u_{k} & \text{if }0\leq i\leq k,\\ 0 & \text{if }i>k. \end{array}\right.$$

Therefore $ux= (u_{k}x_{k})\in \ell_{1}$ whenever $x\in b_{0}^{r,s}( \nabla^{(\alpha)})$, $b_{c}^{r,s}(\nabla^{(\alpha)})$ or $b_{\infty }^{r,s}(\nabla^{(\alpha)})$ if and only if $G^{r,s}y\in \ell_{1}$ whenever $y\in c_{0}, c$ or $\ell_{\infty }$. This implies that $u=(u_{k})\in [b_{0}^{r,s}(\nabla^{(\alpha)})]^{\alpha },[b_{c}^{r,s}( \nabla^{(\alpha)})]^{\alpha }$, or $[b_{\infty }^{r,s}( \nabla^{(\alpha)})]^{\alpha }$ if and only if $G^{r,s}\in (c_{0}:\ell _{1})=(c:\ell_{1})=(\ell_{\infty }:\ell_{1})$. We derive by part (i) of Lemma 3.1 that $u=(u_{k})\in [b_{0}^{r,s}(\nabla^{(\alpha)})]^{ \alpha }=[b_{c}^{r,s}(\nabla^{(\alpha)})]^{\alpha } =[b_{\infty } ^{r,s}(\nabla^{(\alpha)})]^{\alpha }$ if and only if

$$\underset{K\in \Gamma }{\sup}\sum_{k}|\sum_{i\in K}{\left(s+r\right)}^{i}\sum_{j=i}^{k}{\left(-1\right)}^{k-j}\left(\begin{array}{c} j\\ i \end{array}\right)\frac{\Gamma (-\alpha +1)}{\left(k-j)!\Gamma (-\alpha -k+j+1\right)}r^{-j}{\left(-s\right)}^{j-i}u_{k}|<\infty ,$$

which yields that $[b_{0}^{r,s}(\nabla^{(\alpha)})]^{\alpha }=[b_{c} ^{r,s}(\nabla^{(\alpha)})]^{\alpha } =[b_{\infty }^{r,s}( \nabla^{(\alpha)})]^{\alpha }=U^{r,s}_{1}$. □

To determine the *β*- and *γ*-duals of the spaces $b_{0}^{r,s}(\nabla^{(\alpha)})$, $b_{c}^{r,s}(\nabla^{(\alpha)})$, and $b_{\infty }^{r,s}(\nabla^{(\alpha)})$, we define the following sets:

$$\begin{aligned}& U_{2}^{r,s}=\biggl\{ u=(u_{k}): \sup _{n\in \mathbb{N}}\sum_{k} \vert u_{n,k} \vert < \infty \biggr\} , \\& U_{3}^{r,s}=\Bigl\{ u=(u_{k}): \lim _{n\rightarrow \infty } u_{n,k} \text{ exists for each } k \in \mathbb{N}\Bigr\} , \\& U_{4}^{r,s}=\biggl\{ u=(u_{k}): \lim _{n\rightarrow \infty }\sum_{k} \vert u _{n,k} \vert =\sum_{k} \Bigl\vert \lim _{n\rightarrow \infty }u_{n,k} \Bigr\vert \biggr\} , \end{aligned}$$

and

$$\begin{aligned}& U_{5}^{r,s}=\biggl\{ u=(u_{k}): \lim _{n\rightarrow \infty }\sum_{k}u_{n,k} \text{ exists}\biggr\} , \end{aligned}$$

where

$$u_{n,k}={\left(s+r\right)}^{k}\sum_{i=k}^{n}\sum_{j=k}^{i}{\left(-1\right)}^{i-j}\left(\begin{array}{c} j\\ k \end{array}\right)\frac{\Gamma (-\alpha +1)}{\left(i-j)!\Gamma (-\alpha -i+j+1\right)}r^{-j}{\left(-s\right)}^{j-k}u_{i}.$$

### Theorem 3.3

(i) $[b_{0}^{r,s}(\nabla^{(\alpha)})]^{ \beta }=U_{2}^{r,s} \cap U_{3}^{r,s}$,
(ii) $[b_{c}^{r,s}(\nabla^{(\alpha)})]^{ \beta }=U_{2}^{r,s} \cap U_{3}^{r,s}\cap U_{5}^{r,s}$,
(iii) $[b_{\infty }^{r,s}(\nabla^{(\alpha)})]^{ \beta }=U_{3} ^{r,s}\cap U_{4}^{r,s}$,
(iv) $[b_{0}^{r,s}(\nabla^{(\alpha)})]^{\gamma }=[b_{c}^{r,s}( \nabla^{(\alpha)})]^{\gamma }=[b_{\infty }^{r,s}(\nabla^{(\alpha)})]^{ \gamma }= U_{2}^{r,s}$.

### Proof

We consider the equality

$$\begin{array}{rcl} \sum_{k=0}^{n}u_{k}x_{k} & = & \sum_{k=0}^{n}u_{k}\left[\sum_{i=0}^{k}{\left(s+r\right)}^{i}\sum_{j=i}^{k}{\left(-1\right)}^{k-j}\left(\begin{array}{c} j\\ i \end{array}\right)\frac{\Gamma (-\alpha +1)}{\left(k-j)!\Gamma (-\alpha -k+j+1\right)}r^{-j}{\left(-s\right)}^{j-i}y_{i}\right]\\ & = & \sum_{k=0}^{n}\left[{\left(s+r\right)}^{k}\sum_{i=k}^{n}\sum_{j=k}^{i}{\left(-1\right)}^{i-j}\left(\begin{array}{c} j\\ k \end{array}\right)\frac{\Gamma (-\alpha +1)}{\left(i-j)!\Gamma (-\alpha -i+j+1\right)}r^{-j}{\left(-s\right)}^{j-k}u_{i}\right]y_{k}\\ & = & {\left(U^{r,s}y\right)}_{n}, \end{array}$$

where $U^{r,s}=(u^{r,s}_{n,k})$ is defined by

$$u_{n,k}^{r,s}=\left\{ \begin{array}{cc} {\left(s+r\right)}^{k}\sum_{i=k}^{n}\sum_{j=k}^{i}{\left(-1\right)}^{i-j}\left(\begin{array}{c} j\\ k \end{array}\right)\frac{\Gamma (-\alpha +1)}{\left(i-j)!\Gamma (-\alpha -i+j+1\right)}r^{-j}{\left(-s\right)}^{j-k}u_{i} & \text{if }0\leq k\leq n,\\ 0 & \text{if }k>n. \end{array}\right.$$

Therefore $ux= (u_{k}x_{k})\in cs$ whenever $x\in b_{0}^{r,s}( \nabla^{(\alpha)})$ if and only if $U^{r,s}y\in c$ whenever $y\in c_{0}$. This implies that $u=(u_{k})\in [b_{0}^{r,s}( \nabla^{(\alpha)})]^{ \beta }$ if and only if $U^{r,s}\in (c_{0}:c)$. We obtain by part (ii) of Lemma 3.1 that $[b_{0}^{r,s}(\nabla^{( \alpha)})]^{ \beta }=U_{2}^{r,s}\cap U_{3}^{r,s}$. The proof can be completed in a similar way by parts (iii), (iv), (v) instead of Part (ii) of Lemma 3.1, so we omit the details. □

### Theorem 3.4

*The spaces* $[b_{0}^{r,s}(\nabla^{(\alpha)})]^{*}$ *and* $[b_{c}^{r,s}( \nabla^{(\alpha)})]^{*}$ *are equivalent to* $\ell_{1}$.

### Proof

Proof follows from Theorem 3.6 of Bişgin [11] and the fact that if *Z* is a Banach space, then $[Z(\nabla^{(\alpha)})]^{*}$ is equivalent to $X^{*}$ [17]. □

## Conclusion

In this paper, we have discussed some results obtained from the matrix domain of the binomial matrix and the difference matrix of fractional order. Our main aim is to generalize the results on the matrix domain of the Euler matrix. It is immediate that our results reduce to the sequence spaces defined in [11, 12, 18, 23, 25, 26].
